# Difficulties in Emotion Regulation Mediate the Association Between Peer Attachment and Problematic Use of Smartphone

**DOI:** 10.1002/brb3.71224

**Published:** 2026-01-28

**Authors:** Zi‐Liang Wang, Yan‐Li Zhang

**Affiliations:** ^1^ Zhenjiang Mental Health Center Zhenjiang Jiangsu China

**Keywords:** adolescents, emotion regulation, multi‐group SEM, peer attachment, problematic use of smartphone

## Abstract

**Background:**

Problematic use of smartphone (PUS) is increasingly prevalent among adolescents and may be influenced by both peer relationships and emotion‐regulation capacities. This study tested whether difficulties in emotion regulation mediate the association between peer attachment and PUS, and whether these pathways differ by gender, age, and school type.

**Methods:**

A cross‐sectional survey recruited 12,099 Chinese adolescents (mean age = 18.93 years, 58% female) through stratified cluster sampling. Participants completed validated measures of PUS, peer attachment, and the Difficulties in Emotion Regulation Scale (DERS). Structural equation modeling (SEM) examined mediation via a latent DERS factor, controlling for demographics. Multi‐group SEM evaluated moderation across gender, age (> 18 vs. ≤ 18), and school (university vs. high school).

**Results:**

Peer attachment was negatively associated with emotion‐regulation difficulties (*β* = −0.296, *p* < 0.001) and with PUS (*β* = −0.144, *p* < 0.001). Emotion‐regulation difficulties positively predicted PUS (*β* = 0.372, *p* < 0.001). The indirect effect (*β* = −0.110, 95% CI [−0.116, −0.104]) confirmed partial mediation. Multi‐group SEM indicated significant moderation (Δ*χ* ^2^ = 76.01–93.70, all *p* < 0.001): the effect of emotion‐regulation difficulties on PUS was stronger among females; the impact of weaker peer attachment on emotion regulation was stronger among younger adolescents; and school type moderated both the path and the direct effect on PUS. Nevertheless, the mediation pathway remained significant in all subgroups.

**Conclusions:**

Emotion‐regulation difficulties partially explain the link between peer attachment and PUS in adolescents. The strength of these pathways varies by gender, age, and school context, highlighting emotion regulation as a promising intervention target.

## Graphical abstract

Peer attachment is associated with problematic use of smartphones among adolescents through both direct and indirect pathways. Lower peer attachment predicts greater difficulties in emotion regulation, which in turn relates to higher problematic smartphone use. The strength of these associations varies by gender, age, and school type.

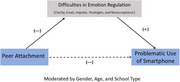

## 1 Introduction

Problematic use of smartphone (PUS) has emerged as a global concern, particularly among adolescents, who increasingly rely on smartphones for communication, entertainment, and academic purposes (Demirci et al. 2015; Xie et al. 2018). Excessive smartphone use can lead to sleep disturbances, emotional dysregulation, academic impairment, and heightened risk for anxiety and depression (Sohn et al. 2021). Understanding the psychosocial mechanisms underlying PUS is critical for designing effective prevention and intervention strategies. Accordingly, it is important to move beyond direct associations and examine the mediating mechanisms that may explain how interpersonal and emotional factors contribute to PUS.

Attachment theory posits that secure interpersonal bonds, particularly with peers during adolescence, provide critical emotional support and foster adaptive coping strategies (Delgado et al. 2022; Flykt et al. 2021; Moretti and Peled 2004). Peer attachment (PA) is characterized by trust, communication, and low alienation (Zhang et al. 2011). Adolescents with higher PA tend to experience greater emotional security and are better able to navigate social and emotional challenges (Muzi et al. 2022). Conversely, lower PA may be associated with heightened emotional distress, which can manifest in maladaptive behaviors such as PUS (Gioia et al. 2021). This suggests that PA may influence PUS indirectly through emotion‐regulation processes, which we discuss next.

Emotion regulation, defined as the ability to monitor, evaluate, and modify emotional responses to achieve goals (Hallion et al. 2018), has been implicated as a key mechanism linking social relationships to behavioral outcomes. Deficits in emotion regulation have been associated with addictive behaviors, including problematic smartphone and internet use (Liang et al. 2021; Nikmanesh et al. 2014). The brief Difficulties in Emotion Regulation Scale (B‐DERS) captures five distinct regulatory dimensions: clarity (awareness and understanding of emotions), goals (ability to engage in goal‐directed behavior despite negative emotions), impulse (control over impulsive actions when distressed), strategies (access to effective regulatory strategies), and nonacceptance (tendency to respond negatively to one's own emotions) (Hallion et al. 2018). Each dimension may contribute differently to PUS, reflecting specific vulnerabilities in emotional processing. Thus, examining both the overall construct and its subdimensions may provide more nuanced insights into how emotion regulation links PA to PUS.

Prior studies suggest that deficits in emotion regulation mediate associations between social factors and problematic digital behaviors (Kuz et al. 2023; Liang et al. 2021). For instance, adolescents with lower PA often exhibit greater emotion‐regulation difficulties, which in turn increase reliance on digital devices as maladaptive coping mechanisms (Liang et al. 2021). However, existing research has rarely examined the distinct contributions of multiple emotion‐regulation dimensions in a large, representative adolescent sample.

In addition, demographic factors such as age, gender, and school context may moderate these pathways. Research has shown that female adolescents are often more vulnerable to problematic smartphone use, particularly when emotional expression and social connection are salient (Cho et al. 2025). Younger adolescents may be especially prone to smartphone overuse due to less mature emotion‐regulation capacities (López‐Mora et al. 2021). Furthermore, school type (high school vs. university) reflects different academic pressures, autonomy, and peer contexts, all of which can shape attachment quality and emotion regulation (Fang et al. 2025). These findings highlight the need for multi‐group approaches that evaluate whether the strength of mediation pathways differs across demographic subgroups.

The present study aimed to investigate (1) the relationships among PA, five dimensions of emotion‐regulation difficulties, and PUS; and (2) whether difficulties in emotion regulation mediate the association between PA and PUS among Chinese adolescents. We hypothesized that (a) higher PA would be associated with fewer emotion‐regulation difficulties (both overall and in specific dimensions) and lower PUS; (b) emotion‐regulation difficulties would positively predict PUS; (c) emotion‐regulation difficulties would partially mediate the association between PA and PUS; and (d) the strength of these mediation pathways would be moderated by gender, age (≤ 18 vs. > 18), and school type (high school vs. university), with stronger effects expected for females, younger adolescents, and students in more academically demanding contexts.

## 2 Methods

Participants were 12,099 adolescents from multiple schools in China, recruited using stratified cluster sampling to ensure representative coverage across grades, regions, and school types. The mean age was 18.93 years (SD = 1.50, range 14.54–24 years), and 58% were female. Age was categorized into two groups for moderation analysis: younger adolescents (≤ 18 years, range 14.54–18.00 years) and older adolescents (> 18 years, range 18.01–24.00 years). To better capture distinctions between educational stages, the sampling framework explicitly included one senior high school and one university from both northern and southern China. Senior high schools were selected to represent the basic education stage, while universities represented the tertiary education stage, thereby enhancing comparability across educational levels and geographical regions. We implemented a rigorous data cleaning procedure to identify and exclude careless or insufficient effort respondents (C/IERs), following established methodologies (Ward and Meade 2023). Five indices were computed to flag low‐quality data: one a priori measure (total response time) and four response‐pattern indicators (longest string of identical responses, psychometric synonyms, intra‐individual response variability, and group normative inconsistency). Each index generated a binary flag (0 = valid, 1 = suspect), which was summed to form a composite C/IER score for each participant. Adopting a conservative criterion, we excluded all participants with a score ≥ 1 from subsequent analyses.

Problematic smartphone use was assessed using the Smartphone Application‐Based Addiction Scale (SABAS) (Peng et al. 2023), a six‐item scale that measures compulsive smartphone engagement and related functional impairment. It is suitable for adolescents and adults and has been validated in Chinese populations (Cronbach's α in this sample = 0.849). PA was assessed using the peer subscale of the inventory of parent and PA (Zhang et al. 2011), which includes 25 items assessing trust, communication, and alienation in peer relationships. It is widely used in adolescent samples and has shown good reliability in Chinese adolescents (Cronbach's α = 0.833). Higher scores indicate stronger PA. Emotion‐regulation difficulties were measured with the B‐DERS (J. Li et al. 2018), a 16‐item scale covering five dimensions: clarity (2 items), goals (3 items), impulse (3 items), strategies (5 items), and nonacceptance (3 items). The scale was developed and validated for Chinese adolescents and young adults (Cronbach's α for total score = 0.865).

Data were analyzed in R (Version 4.2.2) using the Lavaan package for structural equation modeling (SEM) (Rosseel 2012). Bivariate associations among peer attachment, each DERS subscale, and PUS were inspected using Pearson correlation coefficients. We specified a single latent emotion‐regulation difficulties factor (DERS) indicated by the five DERS subscales. Models were estimated with full information maximum likelihood (FIML) to handle missing data and with robust standard errors and test statistics where appropriate (MLR/robust estimation), and all reported parameter standard errors and confidence intervals for indirect effects were obtained via nonparametric bootstrap (*n* = 1000 bootstrap samples) to account for non‐normal sampling distributions of mediation effects (Preacher and Hayes 2008). A random seed was set for reproducibility.

To test the mediation hypothesis, SEM evaluated the direct path from PA to PUS and the indirect path via the latent DERS factor (i.e., PA → DERS → PUS), while controlling for age (in years), gender, and school type. The indirect effect was defined as the product of the PA → DERS (a) and DERS → PUS (b) paths (ind = a × b), and the bootstrap percentile (or bias‐corrected) 95% confidence interval was used to assess significance.

Measurement invariance of the latent DERS factor across groups (gender, age, and school) was examined before testing moderated (group) differences in structural paths. We followed a stepwise invariance procedure: (1) configural invariance (same factor structure across groups), (2) metric invariance (factor loadings constrained equal across groups). If metric invariance held, we proceeded to structural invariance tests. Model comparisons used changes in comparative fit index (ΔCFI ≤ 0.01) as a practically meaningful criterion (Cheung and Rensvold 2002), supplemented by changes in RMSEA.

Moderation (i.e., whether path strengths differed by group) was tested with multi‐group SEM. For each grouping variable, we first fit an unconstrained (free) multi‐group model in which structural regression coefficients were allowed to vary across groups. We then fit a constrained model with the structural regressions (a, b, and c paths) held equal across groups. A significant deterioration of fit in the constrained model (based primarily on ΔCFI > 0.01 and/or a significant *χ* ^2^ difference with appropriate scaling correction) was interpreted as evidence that the strength of the paths differed across groups (i.e., moderation). To complement these omnibus tests and to provide robust inference about group differences in indirect effects, we also performed permutation tests (*n* = 1000 permutations) in which group labels were shuffled and the between‐group difference in the focal parameter (e.g., indirect effect) was recomputed; the empirical permutation distribution provided a *p*‐value for the observed group difference (Jorgensen et al. 2018). All SEM models and permutation procedures used the same preprocessing, covariates, and estimation settings. We report standard model fit indices (CFI, TLI, RMSEA with 90% CI, SRMR) and standardized path estimates. All analyses set a fixed random seed for reproducibility and used 1000 bootstrap samples and 1000 permutations for inferential procedures unless otherwise noted. Statistical significance was set at *α* = 0.05 (two‐tailed). Reported effect sizes are standardized path coefficients and bootstrapped confidence intervals.

## 3 Results

Table 1 presents the descriptive statistics (means and standard deviations) for all key study variables across the entire sample and for each subgroup (gender, age group, and school). Independent samples *t*‐tests revealed significant group differences for most variables. Notably, gender showed significant differences on all variables except DERS_Goals. Age groups (≤ 18 years, range 14.54–18.00 vs. > 18 years, range 18.01–24.00) differed significantly on all DERS subscales and peer attachment, but not on PUS. School differences were significant for all variables except DERS_Nonacceptance.

**TABLE 1:** Descriptive statistics and group differences.

Variable	Overall mean ± SD	Gender (mean ± SD)	*p*	Age group (mean ± SD)	*p*	School (mean ± SD)	*p*
DERS_Clarity	3.67 ± 1.62	1: 3.77 ± 1.65; 2: 3.59 ± 1.59	< 0.001	> 18: 3.62 ± 1.53; ≤ 18: 3.87 ± 1.89	< 0.001	1: 3.64 ± 1.53; 2: 3.81 ± 1.91	< 0.001
DERS_Goals	6.10 ± 2.56	1: 6.10 ± 2.49; 2: 6.11 ± 2.61	0.856	> 18: 6.16 ± 2.51; ≤ 18: 5.89 ± 2.71	< 0.001	1: 6.23 ± 2.50; 2: 5.67 ± 2.70	< 0.001
DERS_Impulse	4.67 ± 2.08	1: 4.72 ± 2.12; 2: 4.63 ± 2.05	0.019	> 18: 4.62 ± 2.00; ≤ 18: 4.85 ± 2.34	< 0.001	1: 4.63 ± 1.99; 2: 4.81 ± 2.36	< 0.001
DERS_Strategies	8.51 ± 3.57	1: 8.67 ± 3.69; 2: 8.39 ± 3.47	< 0.001	> 18: 8.41 ± 3.40; ≤ 18: 8.90 ± 4.09	< 0.001	1: 8.45 ± 3.39; 2: 8.75 ± 4.15	0.0005
DERS_Nonaccept	5.31 ± 2.20	1: 5.45 ± 2.24; 2: 5.19 ± 2.15	< 0.001	> 18: 5.28 ± 2.12; ≤ 18: 5.43 ± 2.45	0.005	1: 5.31 ± 2.11; 2: 5.33 ± 2.47	0.668
PUS_Sum	18.87 ± 5.37	1: 19.24 ± 5.29; 2: 18.57 ± 5.41	< 0.001	> 18: 18.91 ± 5.27; ≤ 18: 18.73 ± 5.70	0.136	1: 19.04 ± 5.19; 2: 18.27 ± 5.91	< 0.001
PA_Sum	88.79 ± 12.14	1: 90.99 ± 11.92; 2: 87.00 ± 12.03	< 0.001	> 18: 88.95 ± 11.92; ≤ 18: 88.23 ± 12.90	0.01	1: 89.01 ± 11.79; 2: 88.02 ± 13.27	0.0004

*Note*: Gender, 1 = male; 2 = female; school, 1 = university; 2 = high school. Age groups are defined as > 18 and ≤ 18 years.

Abbreviations: DERS, Difficulties in Emotion Regulation Scale; PA, peer attachment; PUS, problematic use of smartphone.

These pervasive differences underscore the necessity of controlling for these demographic variables in the subsequent structural equation models to isolate the unique relationships between the primary constructs of interest.

Table 2 displays the bivariate correlations between the study variables. As hypothesized, the PA_Sum score was significantly and negatively correlated with all facets of emotion dysregulation (DERS subscales, |*r*| = 0.175 to 0.269, *p* < 0.001) and with PUS_sum, *r* = −0.231, *p* < 0.001). Conversely, all DERS subscales were positively and significantly intercorrelated (*r* = 0.551 to 0.811, *p* < 0.001), demonstrating strong internal consistency for the latent construct. Furthermore, each DERS subscale was positively correlated with PUS_Sum (*r* = 0.320 to 0.378, *p* < 0.001). This pattern of correlations provides strong preliminary support for the proposed mediation model, confirming that the constructs are related in the expected directions.

**TABLE 2:** Correlations among key study variables.

Variable	1	2	3	4	5	6	7
1. PA_Sum	—						
2. DERS_Clarity	−0.231***	—					
3. DERS_Goals	−0.175***	0.579***	—				
4. DERS_Impulse	−0.239***	0.552***	0.617***	—			
5. DERS_Strategies	−0.269***	0.689***	0.731***	0.742***	—		
6. DERS_Nonaccept	−0.227***	0.641***	0.692***	0.677***	0.811***	—	
7. PUS_Sum	−0.231***	0.320***	0.351***	0.331***	0.378***	0.360***	—

Abbreviations: DERS, Difficulties in Emotion Regulation Scale; PA, peer attachment; PUS, problematic use of smartphone.

The full sample model demonstrated an excellent fit to the data (CFI = 0.984, TLI = 0.975, RMSEA = 0.049, SRMR = 0.020), exceeding conventional thresholds for good fit (CFI/TLI > 0.95, RMSEA 0 < 0.06, SRMR < 0.08). Table 3 presents the standardized path coefficients for the full sample structural equation model. Supporting our hypothesis, poorer PA significantly predicted higher emotion dysregulation (DERS latent factor) (*β* = −0.296, *p* < 0.001; a path). In turn, higher emotion dysregulation significantly predicted greater problem mobile phone use (PUS) (*β* = 0.372, *p* < 0.001; b path). The bootstrapped indirect effect (a*b) was significant (*β* = −0.110, 95% CI [−0.116, −0.104], *p* < 0.001), confirming that emotion dysregulation mediates the relationship between PA and problem mobile phone use. The direct effect of PA on PUS remained significant (*β* = −0.144, *p* < 0.001; c′ path), indicating partial mediation. All control variables (age, gender, and school) had significant effects on either DERS, PUS, or both, justifying their inclusion.

**TABLE 3:** Standardized path coefficients for the full sample SEM.

Path	*β*	*p*	95% CI lower	95% CI upper
Direct effects				
PA → DERS (a path)	−0.296	< 0.001	−0.31	−0.282
DERS → PUS (b path)	0.372	< 0.001	0.357	0.387
PA → PUS (c′ path)	−0.144	< 0.001	−0.158	−0.13
Indirect effect				
PA → DERS → PUS (a*b)	−0.11	< 0.001	−0.116	−0.104
Control variables on DERS				
Age → DERS	−0.094	< 0.001	−0.112	−0.075
Gender → DERS	−0.095	< 0.001	−0.111	−0.078
School → DERS	−0.073	< 0.001	−0.087	−0.058
Control variables on PUS				
Age → PUS	−0.043	< 0.001	−0.062	−0.024
Gender → PUS	−0.081	< 0.001	−0.096	−0.065
School → PUS	−0.112	< 0.001	−0.128	−0.095

*Note*: All control variable paths were statistically significant (*p* < 0.001).

Abbreviations: CI = confidence interval; DERS = Difficulties in Emotion Regulation Scale; PA = peer attachment; PUS = problematic use of smartphone.

The multi‐group models, which tested measurement and structural paths across different levels of gender, age, and school, also all exhibited excellent fit (CFI range = 0.985 to 0.992, RMSEA range = 0.038 to 0.052). The strong fit of these models indicates that the hypothesized structure is robust and appropriate for testing across diverse subgroups.

To examine the moderating effects of gender, age, and school, we conducted multi‐group analyses. As shown in Table 4, the chi‐square difference tests were all significant (for gender: Δ*χ* ^2^(7) = 76.01, *p* < 0.001; for age group: Δ*χ* ^2^(7) = 85.17, *p* < 0.001; for school: Δ*χ* ^2^(7) = 93.70, *p* < 0.001). This indicates that constraining the structural paths to be equal across groups resulted in a significantly worse model fit. Therefore, the structural paths differed significantly across groups, confirming that gender, age, and school each significantly moderated at least one path within the proposed model. The moderating effect was primarily observed on the b path (DERS → PUS). Specifically, the unstandardized coefficient for the b path was significantly higher for females (Group 2; *b* = 1.864) than for males (Group 1; *b* = 1.673). This indicates that the effect of emotion dysregulation on problematic phone use was stronger in the female group. For females, emotional difficulties are more likely to lead to excessive mobile phone use for regulation or escapism. The moderating effect was primarily observed on the a path (PA → DERS). The coefficient for this path was more negative for minors (≤ 18 years; *b* = −0.031) than for adults (> 18 years; *b* = −0.026). This suggests that the negative impact of poorer PA on emotion dysregulation was stronger among minors. The emotional well‐being of adolescents is more closely tied to the family system and the quality of the parent–child relationship. For the a path, the coefficient was more negative for university (*b* = −0.027) than for high school (*b* = −0.023), indicating that the protective effect of PA against emotion dysregulation was stronger in the environment of university.

**TABLE 4:** Results of multi‐group invariance testing and indirect effects.

Group variable	*χ* ^2^(df)	*p*	Moderated path	Group 1	Group 2
Gender	76.01(7)	< 0.001	b (DERS → PUS)	1.673 (male)	1.864 (female)
			a (PA → DERS)	−0.027 (male)	−0.031 (female)
Age group	85.17(7)	< 0.001	a (PA → DERS)	−0.026 (adults)	−0.031 (minors)
			Control: gender → DERS	−0.130 (adults)	−0.110 (minors)
School	93.70(7)	< 0.001	a (PA → DERS)	−0.027 (university)	−0.023 (high school)
			Control: school → PUS	−1.57 (university)	−2.48 (high school)
			Control: age → DERS	−0.075 (university)	−0.043 (high school)

Abbreviations: DERS, Difficulties in Emotion Regulation Scale; PA, peer attachment; PUS, problematic use of smartphone.

Furthermore, the direct effect of the school environment on PUS (School → PUS) also differed significantly. The negative direct effect was much stronger for high school (*b* = −2.478) than for university (*b* = −1.565). This implies that high school may possess unique environmental factors (e.g., stricter regulations, distinct school culture) that more directly reduce student phone use, independent of the psychological pathway of PA and emotion dysregulation. It is crucial to note that despite the moderation of path strengths, the core indirect effect (PA → DERS → PUS) remained statistically significant within every subgroup. This demonstrates that the mediating mechanism through emotion dysregulation is a robust and pervasive mechanism, yet its strength varies meaningfully depending on an individual's gender, age, and environmental context.

## 4 Discussion

This study provides empirical evidence that emotion‐regulation difficulties partially mediate the association between PA and problematic smartphone use in Chinese adolescents. The large, representative sample enhances the robustness of these findings. Consistent with attachment theory (Delgado et al. 2022; Flykt et al. 2021), adolescents with stronger PA reported fewer difficulties across all five emotion‐regulation dimensions. Trust and effective communication with peers likely facilitate adaptive coping strategies and reduce reliance on maladaptive behaviors such as excessive smartphone use (Muzi et al. 2022). Conversely, adolescents with lower PA may experience emotional insecurity, leading to heightened vulnerability to PUS. Beyond overall mediation, multi‐group analyses revealed significant demographic moderators. Gender moderated the pathway from emotion regulation to problematic use, with a stronger association among females. This aligns with evidence that adolescent girls are more likely to use smartphones for emotional coping and social connection, making them more vulnerable when regulatory capacities are impaired. Age moderated the influence of attachment on emotion regulation, with a stronger impact among younger adolescents (≤ 18 years) who are more embedded in peer systems and depend heavily on external emotional support. School type exerted both direct and indirect effects, reflecting differences in academic demands, institutional rules, and peer contexts between educational stages. These results underscore the need for tailored interventions considering demographic and contextual factors.

Each DERS dimension contributed uniquely to problematic smartphone use. Difficulty in emotional clarity may impair awareness of distress and precipitate reactive smartphone engagement as a form of self‐soothing (Ding et al. 2022; Gündoğmuş et al. 2021; Jin et al. 2023). Challenges in goal‐directed behavior suggest that adolescents may struggle to pursue tasks when distressed, turning to smartphone use as avoidance (Akbari 2017; Gu 2022; Lei et al. 2025). Impulse control difficulties directly increase the likelihood of compulsive checking behaviors (Fabio et al. 2022; Pérez de Albéniz Garrote et al. 2021). Limited access to regulatory strategies indicates a deficit in adaptive coping, amplifying reliance on digital distractions (Extremera et al. 2019). Nonacceptance of emotional responses may intensify negative affect and drive smartphone use to mitigate emotional discomfort (Gioia et al. 2021). These findings support previous research linking specific emotion‐regulation deficits to behavioral addictions. It is important to note that the cross‐sectional design precludes causal inference. The relationships among attachment, emotion regulation, and PUS are likely bidirectional and dynamic over time. For example, excessive smartphone use may itself impair emotion regulation and erode peer relationships (Hu et al. 2025). Future longitudinal studies are needed to clarify temporal precedence and potential feedback loops.

Although emotion‐regulation difficulties partially mediated the PA‐PUS link, a direct effect remained. This suggests additional mechanisms, such as social modeling, normative peer behaviors, and perceived social support, may independently influence smartphone use (Marino et al. 2020; Xu et al. 2023). The interplay between social environment and individual regulatory capacities highlights the multifactorial nature of adolescent PUS. Specifically, perceived social norms and peer pressure can shape usage patterns, as adolescents often adopt behaviors accepted within their social circle to gain acceptance and avoid exclusion (Van Woudenberg et al. 2019). Furthermore, inadequate social support, particularly from family and peers, may drive compensatory use of smartphones for emotional comfort and relational connection, consistent with the Compensatory Internet Use Theory (Arrivillaga et al. 2022). This pattern is especially prominent when psychological needs remain unmet in offline environments (D. Li et al. 2023). In addition, social modeling and identity‐seeking motivations contribute to problematic use; adolescents frequently observe and imitate the smartphone‐related behaviors of influential others, while also utilizing digital platforms to explore and express their self‐identity, particularly during a developmental stage characterized by heightened self‐concept uncertainty (Shaw et al. 2016). These social and cognitive mechanisms operate alongside rather than through emotion regulation pathways, highlighting the multifactorial nature of problematic smartphone use (Valkenburg et al. 2022). Effective interventions should therefore not only focus on improving emotional regulation but also address peer influences, enhance perceived social support, and encourage the fulfillment of psychological needs in offline contexts (Dancoine and Gentina 2018).

Beyond the overall mediation model, our multi‐group analyses highlight important demographic moderators. First, gender significantly altered the pathway from emotion regulation to problematic smartphone use, with the association being stronger among females. This finding is consistent with evidence that adolescent girls are more likely to use smartphones for emotional coping and social connection, making them more vulnerable when regulatory capacities are impaired (Cheng et al. 2024). Second, age moderated the influence of attachment on emotion regulation. The impact of poor PA on regulatory difficulties was stronger among younger adolescents (≤ 18), who are still highly embedded in family and peer systems and depend more heavily on external sources of emotional support. Developmental studies similarly suggest that regulatory skills consolidate with age, partly buffering the influence of attachment quality in older youth (Theurel and Gentaz 2018). Finally, school type exerted both direct and indirect effects. Compared to university students, high school students showed a stronger protective effect of attachment against dysregulation, but also a stronger direct negative effect of school environment on smartphone use. This may reflect differences in academic demands, institutional rules, and peer contexts between high school and university settings (Rudolf and Kim 2024). Together, these results underscore that demographic and contextual factors shape how attachment and emotion regulation contribute to adolescent PUS, highlighting the need for tailored interventions.

The stronger mediation pathway among females aligns with cross‐cultural evidence that girls are more likely to internalize emotional distress and use relational coping strategies, including digital communication, for emotion regulation (Do et al. 2025). In many societies, girls are socialized to be more emotionally expressive and interpersonally oriented, which may increase their reliance on smartphones for social support when facing emotional challenges (Cheng et al. 2024). For younger adolescents (≤ 18 years), the stronger link between PA and emotion regulation may reflect developmental dependence on peers for identity formation and emotional scaffolding, a pattern observed across diverse cultural contexts (Sahi et al. 2023). The school‐type moderation suggests that structural factors such as academic pressure and autonomy influence how attachment and regulation translate into behavior, a phenomenon also noted in comparative educational research (Rudolf and Kim 2024).

Taken together, these findings highlight that while the mediating mechanism of emotion dysregulation is robust across all subgroups, its strength and consequences are contingent on adolescents’ demographic and contextual backgrounds. Tailored interventions should therefore account for these differences. For example, by focusing on emotional coping strategies in girls, strengthening peer relationships in younger adolescents, and adapting school‐level policies that recognize the unique challenges of high school versus university environments. Limitations include cross‐sectional design, precluding causal inference, and reliance on self‐report measures, which may be subject to bias. Future longitudinal studies and multimodal assessments are warranted to confirm temporal dynamics and generalizability beyond Chinese adolescents. School or community‐based programs targeting emotion regulation could be integrated into existing mental health curricula. For instance, mindfulness‐based interventions could help improve emotional clarity and nonacceptance; cognitive‐behavioral modules could address impulse control and goal‐directed behavior; and peer‐led support groups could foster secure attachment and model adaptive coping (Lindenberg et al. 2022). For high school students, structured digital literacy workshops combined with emotion‐regulation skill‐building may be particularly effective, whereas university interventions might focus on promoting offline social engagement and stress management strategies (Olson et al. 2022). Furthermore, interventions that foster peer trust and communication could indirectly reduce PUS by enhancing emotion regulation.

## 5 Conclusion

In conclusion, emotion‐regulation difficulties partially mediate the association between PA and problematic smartphone use among Chinese adolescents. Our findings indicate that a “one‐size‐fits‐all” intervention approach is unlikely to be effective. Instead, prevention efforts should be tailored to demographic and contextual factors: for example, emotion‐regulation training may be particularly beneficial for girls, peer‐support programs may be especially important for younger adolescents, and school‐level policies should consider the distinct environments of high schools versus universities. Targeted interventions that simultaneously strengthen peer relationships and build emotion‐regulation skills offer a promising pathway to reducing problematic smartphone use and promoting healthier adolescent development.

## Author Contributions

Zi‐Liang Wang analyzed the data and wrote the first draft of the manuscript. Yan‐Li Zhang contributed to conceptualization, supervision, writing – review and editing. All authors have approved the final manuscript.

## Funding

This research was supported by Zhenjiang Science and Technology Innovation Fund project (JC2024042); Jiangsu Province Natural Science Youth Foundation Project (BK20240506); Jiangsu Provincial Administration of Traditional Chinese Medicine General Project (MS2024122); General Project of Jiangsu Provincial Health Commission (M2024011); The funders had no role in the design and conduct of the study; collection, management, analysis, and interpretation of the data; preparation, review, or approval of the manuscript; and decision to submit the manuscript for publication.

## Ethics Statement

Written informed consent was obtained from both participants and their guardians. All procedures received ethical approval from the Medical Ethics Committee of Zhenjiang Mental Health Center (2023K18) and complied with the Declaration of Helsinki.

## Conflicts of Interest

The authors declare no conflicts of interest.

## Data Availability

Data are available upon reasonable request from the corresponding author at zyl_317@163.com.
